# Committee of the American Medical Association on Epidemics of Illinois, Missouri, Iowa, and Wisconsin

**Published:** 1852-09

**Authors:** 


*Committee of the American Medical Association on Epidemics of Illinois, Missouri, Iowa, and Wisconsin.*

We have received the circulars of this Committee, addressed to the physicians of the States enumerated above, requesting detailed accounts of all the epidemics that have prevailed within the last few years under their observation.

As many of our subscribers will not receive these circulars, on account of the great difficulty of securing a list of names by the chairman of the committee, we hope all will furnish the committee with any information they may possess upon the subject; that we may have a full account of the epidemics of these States, in a form that will be acceptable for future reference and study.

Those physicians who furnish accounts from Missouri are requested to send to Dr. Thos. Reyburn, St. Louis; those from Illinois, to Dr. John Evans, Chicago; and those from Iowa and Wisconsin, to Dr. John F. Sandford, Keokuk, Iowa.

The information thus furnished will be duly credited in the body of the report of the committee. Such accounts as are sent in by or before the 1st of March next will be in time. E.

